# Effective Monitoring of Small River Basins

**DOI:** 10.1100/tsw.2002.160

**Published:** 2002-04-13

**Authors:** W. Symader, R. Bierl, A. Krein

**Affiliations:** Hydrological Department, University of Trier, FB VI-Hydrologie, D-54286 Trier, Germany

## Abstract

As the transport of many pollutants occurs during high floods monitoring programs must focus on these intermittent events. In small rivers the pollutants start their travel as short pulses often associated with fine particles, but disperse on their way downstreams. Therefore the chemical data of a flood event are only representative of a small part of the basin adjacent to the monitoring station. This is usually not taken into account by evaluating water quality data.

## INTRODUCTION

In small river basins, the monitoring of water quality that includes particle-associated contaminants encounters several problems because dry weather flow is of much less importance than intermittent events. To begin with, both particles and particle-associated contaminants are mobilised mainly by events, so sampling strategies based on regular intervals are not adequate. Second, the amount of particle-associated contaminants that are found during an event is supply controlled, which means that identifying the sources is one of the prerequisites of a successful river management. Third, about 95% of runoff, particles, or contaminants come from 5% of a basin. As the chemical characteristics of some of the sources are not constant over time, their temporal patterns have to be understood. Fourth, the role of river bottom sediments is poorly understood. Riverbeds store material only temporarily, which means that they act as sources and sinks. Fifth, even in small basins there is a kinematic wave effect. So hydrographs and chemographs may represent different stages of the event, which leads to the question how representative a sample is.

## MATERIAL AND METHODS

In the region of Trier, four small basins with basin sizes ranging from 1 to 300 $km^2$ have been investigated since 1988. All basins are situated in a mountainous region, but differ in bedrock and the degree of human impact. The results presented here come from the Kartelbornsbach (3 $km^2$), a limestone basin, and the Olewiger Bach (30 $km^2$), which drains a schist and shale area. The advantage of focussing on small basins is that the best approach to diminish water pollution is to improve the situation at the pollution sources. Sampling covered as many aspects as possible, including the hydrologic conditions, particle characteristics, dissolved and suspended major ions, heavy metals, and selected organic contaminants. If there is no preinformation, hand sampling is recommended. Later on, automatic sampling can be used.

## RESULTS

Building up a monitoring system consists of several steps. Daily samples can give some information about the seasonal cycle, but the focus must be on the intermittent events. The sampled flood waves showed a wide variation of flood responses to rain events. In order to condense this information we looked for typical response patterns, the deviations from these patterns, and the reasons for the deviations. The typical response of a small basin to a one-peaked summer event is shown in Fig. 1. The event starts with a small preflush coming from impervious surfaces and the sewer system that is followed by the main event. The chemograph shows a succession of three peaks for the elements calcium (Ca), manganese (Mn), and iron (Fe). Deviations from this response type occur when there is not sufficient precipitation to develop the complete pattern. Comparisons of several events show that with decreasing precipitation, first the peak of Fe and then that of Mn does not appear, whereas Ca is mobilised even by small amounts of rain. The water content of the soil in the valley bottom is responsible for the time lag between the peaks. Speed and direction of a moving cloud cell can modify the typical basic pattern of response as well. Against this first framework of understanding, the basic patterns and possible deviations, peaks of organic contaminants that behave more or less irregularly, can be evaluated.

Winter events do not show a clear response type, as the typical precipitation event is long lasting with varying intensities. This results often in a multipeaked response, where single events cannot be discriminated. Snow melts and rain on frozen grounds are special cases. But winter floods mostly dilute the concentrations of pollution and are no problem for the biocoenosis.

The next step is the identification of both type and location of the sources that govern the input of pollutants into the river system. Investigations on the behaviour of artificial flood waves that were released from a water works station showed that the kinematic wave effect is not restricted to large river systems, but can be observed in small basins too[1]. In relation to the size of the basin or the river, the wave and the water body have been totally decoupled after 3 km in the Olewiger Bach leading to a flood wave that consists totally of old river water. These findings have tremendous consequences. On the one hand, a flood wave that is monitored is representative of an area of only up to 3 km upstream. As it is impossible to have gauging stations at intervals of 3 km, the relationship between basin characteristics and flood response must be understood. On the other hand, the kinematic wave effect means that any striking chemical variations that are observed within the flood response must come from a region up to 3 km upstream of the sampling point, which decreases the number of potential pollution sources considerably. Fig. 2 shows an example, where only two farms could be considered as the potential cause of this double-peaked pollution signal.

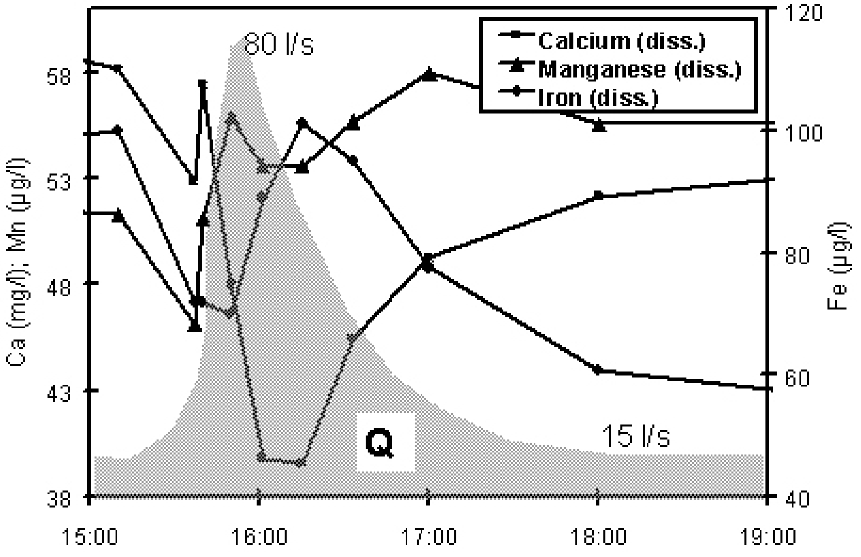

**FIGURE 1**. Chemical variations of Ca, Mn, and Fe during a single peaked flood wave (Kartelbornsbach, 29th of October, 1996) as a typical example of flood response of a small mountainous basin to summer showers.

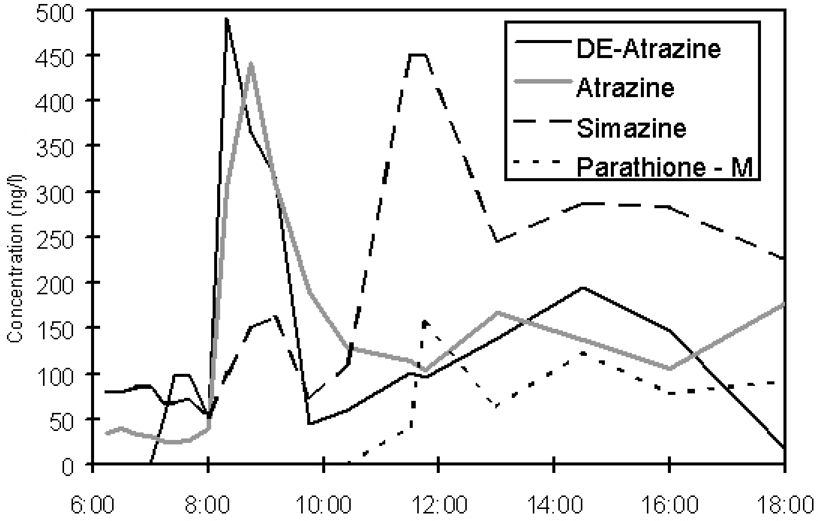

**FIGURE 2**. Four pesticides during a flood event in a small mountainous river show two peaks in their chemographs that are caused by two dominant pollution sources (example Olewiger Bach).

The analysis of the water quality response to precipitation events can help to find sound hypotheses about the sources, but it is no proof. Therefore external evidence is needed. In the case of point sources this is quite easy, as they can be sampled directly. Concerning diffuse sources longitudinal profiles of channel sections can help. The source of the concentration peaks of Ca, Mn, and Fe (Fig. 1) was riparian water entering the river through macropores. This was proven by sampling water from macropores after rain events. High concentrations of pesticides from at least one of the farms could be found in surface water coming from rural pathway system.

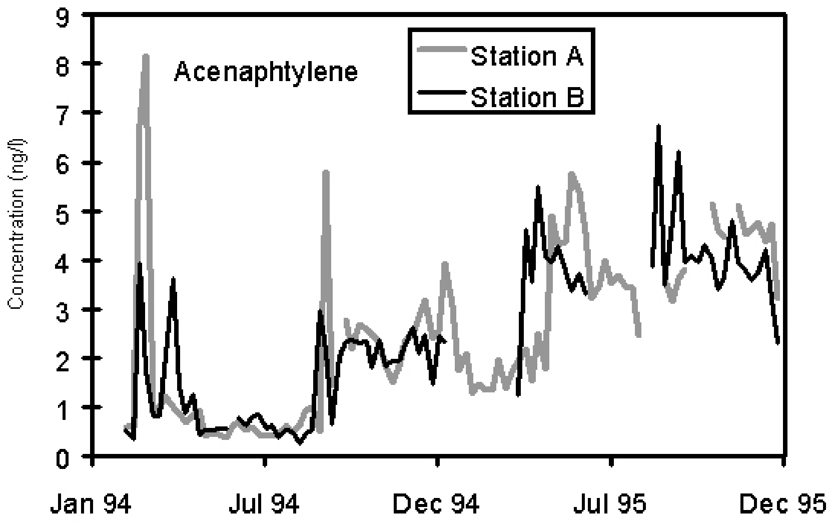

**FIGURE 3**. Time series of weekly samples of acenaphtylene in river bottom sediments taken at two stations of the Kartelbornsbach.

Pollutants that pass the river system travelling as fast pulses associated with suspended particles can be detected by monitoring flood events, when the internal structure of the flood response is understood. However, a lot of substances are released during low flow conditions, when the particles deposit after a short travelling distance. They are temporarily stored in the river bottom sediments and can be remobilised at any time. Neither the storing process nor the remobilisation is understood very well. In mountainous regions there is a dynamic equilibrium between the deposition of suspended particles and the remobilisation of fine particles [2]. Pollutants that are stored just on the surface of the riverbed are transported very rapidly downstream, as Fig. 3 shows. Concentrations of PAHs measured at two stations in a small river occur either simultaneously or with a time lag of no more than 1 week. Remobilisation of deeper layers occurs mostly at the beginning of the rising limb of flood waves in autumn or winter. During extreme events erosion can even reach the bedrock. The river bottom sediment acts as a second agent of fluvial transport and behaves mainly independent of the water body. If the dynamic of the river bottom is understood in a basin, most of the pollution sources can be tracked down using the information of flood response and river bottom sediments simultaneously.

## CONCLUSIONS

The central problem of a monitoring concept in small basins is the intermittent events and the identification of those few potential sources within the basin that are connected to the river system. The monitoring approach consists of four steps:

1. Looking for a typical response type to understand the internal structure of a flood wave;
2. Identification of both the type and location of potential pollution sources (knowledge about the kinematic wave effect helps);
3. Verification of these sources by independent research;
4. Monitoring bottom sediments.
